# Combination of Intravitreal Bevacizumab and Topical Dorzolamide versus Intravitreal Bevacizumab Alone for Diabetic Macular Edema: A Randomized Contralateral Clinical Trial

**DOI:** 10.1155/2020/6794391

**Published:** 2020-01-16

**Authors:** Farhad Fazel, Hossein Nikpour, Mohsen Pourazizi

**Affiliations:** ^1^Isfahan Eye Research Center, Department of Ophthalmology, Isfahan University of Medical Sciences, Isfahan, Iran; ^2^Pediatric Inherited Diseases Research Center, Research Institute for Primordial Prevention of Non-Communicable Disease, Isfahan University of Medical Sciences, Isfahan, Iran

## Abstract

**Purpose:**

To evaluate the efficacy of three intravitreal bevacizumab (IVB) injections versus the same combined with 2% of topical dorzolamide in the treatment of diabetic macular edema (DME).

**Methods:**

In this randomized double-masked clinical trial, 32 eyes of 16 treatment-naive patients with bilateral DME were enrolled. The eyes were randomly assigned to receive three monthly injections of IVB (1.25 mg) plus topical dorzolamide 2% twice daily or IVB (1.25 mg) plus topical artificial tear twice daily. Best-corrected visual acuity (BCVA) was the primary outcome of the study followed by the central macular thickness (CMT) and central macular volume (CMV) as the secondary outcomes.

**Results:**

Mean BCVA changes were insignificant in both groups. It changed from 0.21 ± 0.08 logMAR at baseline to 0.23 ± 0.09 (*P*=0.24) in the combination group and from 0.18 ± 0.09 logMAR to 0.21 ± 0.09 (*P*=0.11) in the IVB alone group, at 3 months, respectively. Changes in mean CMT and CMV were significant in both groups. However, the difference between the groups was not significant at all the visits. In the study, no major ocular complication or systemic side effects were noted regarding IVB or topical dorzolamide.

**Conclusion:**

This randomized contralateral clinical trial demonstrated that adjuvant topical dorzolamide with IVB injection had no additional effects on IVB in the treatment of DME over a three-month course. This trial is registered with the Iranian Registry of Clinical Trials under the registration code IRCT20131229015975N5.

## 1. Introduction

Macular edema (ME) occurs in a variety of pathologic conditions, including diabetic retinopathy, central and branch retinal vein occlusions, uveitis, retinitis pigmentosa (RP), and after surgery [1–3].

Diabetic macular edema (DME) is one of the major causes of visual loss in patients with diabetes mellitus [4, 5] and it has a huge impact on the life quality of patients [6]. The treatment of DME still remains controversial [4, 7]. To date, anti-vascular endothelial growth factor (anti-VEGF) drugs (e.g., ranibizumab, bevacizumab, and VEGF Trap-Eye) have been the best treatment for DME. However, there is a major concern in patients who are resistant to anti-VEGF and those who have recurrent or chronic DME for which anti-VEGF therapy is often unsatisfactory [4, 8, 9].

Dorzolamide, which is classified in carbonic anhydrase inhibitors (CAIs), is widely used to treat glaucoma [10]. Recently, there has been a clinical interest in the use of dorzolamide to treat macular edema with various etiologies [11–16]. In a similar way, topical dorzolamide may be effective in the treatment of DME. It had been hypothesized that, in ME with any underlying pathology, dorzolamide may decrease the aqueous production and the outflow which could subsequently slow the clearance of intravitreous drugs [17]. Thus, the combination of the topical dorzolamide and intravitreal bevacizumab (IVB) seems to have a beneficial effect [17].

To date, there have been no studies regarding the clinical efficacy of dorzolamide eye drop on DME, so the current study would be valuable to optimize the treatment of ME on diabetic patients. The clinical implications of our study are relevant to eyes with DME which may be resistant to anti-VEGF therapy. Accordingly, the present study aimed to determine whether or not adding dorzolamide to conventional treatment of DME would be effective in reducing macular edema in patients with DM. In this randomized contralateral clinical trial, we aimed to evaluate the possible additional effects of dorzolamide to IVB in the treatment of DME.

## 2. Material and Methods

### 2.1. Ethics and Participants

This randomized clinical trial was conducted on 32 eyes of 16 treatment-naive diabetic patients with bilateral DME who were referred to the Feiz Eye Hospital, a referral ophthalmology center affiliated to Isfahan University of Medical Sciences (IUMS), Iran, between April 2017 and April 2018. Informed consent was obtained from each patient before the initiation of the study. The study protocols were approved by the IUMS Research Ethics Committee, Iran. The study was registered at the Iranian Registry of Clinical Trials (registration number: IRCT20131229015975N5). 16 diabetic patients who had bilateral DME according to the Early Treatment Diabetic Retinopathy Study (ETDRS) criteria were enrolled in the study [18]. They had best-corrected visual acuity (BCVA) between 20/50 and 20/200, central macular thickness (CMT) greater than 300 microns as measured by an optical coherence tomography (OCT), and less than 25% differences between eyes. The exclusion criteria were pregnancy, breastfeeding, history of allergy to the study medications, significant macular diseases (e.g., foveal atrophy, foveal scar, etc.), other causes of macular edema (e.g., uveitis or other ocular inflammatory diseases, neovascular glaucoma, epiretinal membrane, etc.), history of ocular surgery, coexistence of ocular diseases including glaucoma, significant media opacity, monocularity, severe comorbidities, uncontrolled diabetes mellitus, uncontrolled hypertension, history of cerebrovascular accident, opaque media, and active ocular infection.

The withdrawal criteria included not attending follow-up visits, receiving other topical or systemic agents during the study, and intolerable side effects.

### 2.2. Treatment Protocol

Three monthly injections of 1.25 *μ*g of bevacizumab (Avastin; Genentech, Inc., South San Francisco, CA, USA) were performed on both eyes of all patients. Intravitreal injections were conducted under the sterile conditions with topical anesthesia and insertion of a lid speculum with a 30-gauge needle through the supratemporal quadrant.

The tested product was dorzolamide 2% (Sina-Daru Pharmaceutical Co., Tehran, Iran) packaged in droppers as against the placebo, artificial tear drops, Tearlose@ (Sina-Daru Pharmaceutical Co., Tehran, Iran) packaged in identical droppers. Each subject received both products with the dose of one drop twice daily on each eye. Each eye was randomly assigned to receive one group of the medication. All preparations were individually made for each patient, and patients and investigators were kept blinded to the randomized allocation and details of the series of the medications throughout the study.

### 2.3. Clinical Assessment

During the baseline examinations, the patients underwent ophthalmologic examinations including measurement of best-corrected visual acuity (BCVA) using Snellen chart, measurement of intraocular pressure (IOP), anterior segment slit-lamp and fundus examination, and measurement of central macular thickness (CMT) and Central Macular Volume (CMV) by spectral domain optical coherence tomography (SD-OCT) (Spectralis; Heidelberg Engineering, Heidelberg, Germany). Such examinations were repeated 1 or 2 months after the first intervention to assure that potential adverse effect visits were planned weekly.

### 2.4. Outcome Measurement

Change in the BCVA was the primary outcome and changes in CMT and CMV were secondary. The potential injection-related complications (e.g., ocular hypertension, anterior chamber reaction, lens opacity progression, and traumatic cataract) were evaluated at each postinjection visit.

### 2.5. Statistical Analysis

Snellen acuities were converted to logarithm of the minimum angle of resolution (logMAR) equivalent values. Data analysis was performed using SPSS (version 18.0) software (Statistical Procedures for Social Sciences, Chicago, IL). The variables were expressed as mean ± standard deviation (SD). Between-group and within-group analyses were performed using Mann–Whitney and Friedman tests, respectively. *P* < 0.05 was considered statistically significant.

## 3. Results

Sixteen patients (32 eyes) completed the study per protocol. The mean age of the participants was 62 ± 13 years. A total of 6 patients (37.5%) were male.

### 3.1. Within-Group Analysis after Intervention

Table 1 reveals the difference in the BCVA within the groups at baseline and during the study.

Compared with the baseline values, the mean BCVA was improved at 1 and 2 months in both groups; however, these improvements did not reach a significant level in the within-group analyses. There was a significant decrease regarding CMV and CMT during the study compared with the baseline in both groups. The corresponding *P*values for CMV were as follows: combination group, *P*=0.002; and control group, *P*=0.039 (Table 2).

The corresponding *P* values for CMT were as follows: combination group, *P*=0.013; and control group, *P*=0.003 (Table 3).

### 3.2. Between-Group Analysis after Intervention

**Figure 1:**
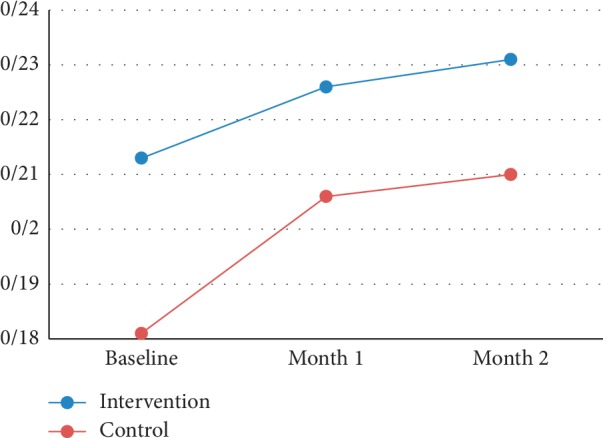
Trend of BCVA change in both groups during the study.

The differences in BCVA changes between the groups were not significant at 1 and 2 months (*P*=0.53 and *P*=0.64, respectively) (Table 1).  Figure 1 shows the trend of BCVA change in both groups during the study.

**Figure 2:**
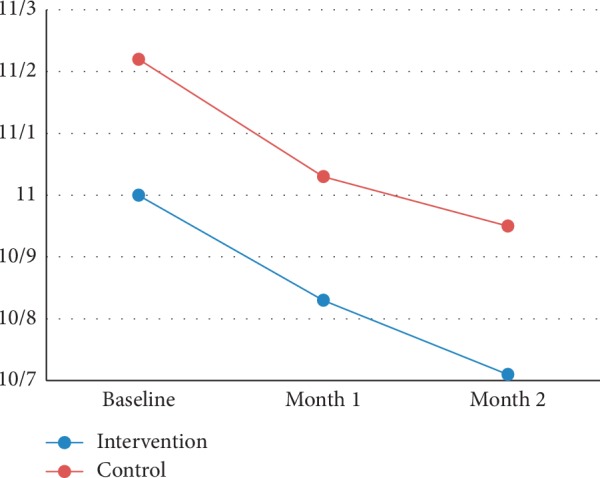
Trend of CMV change in both groups during the study.

**Figure 3:**
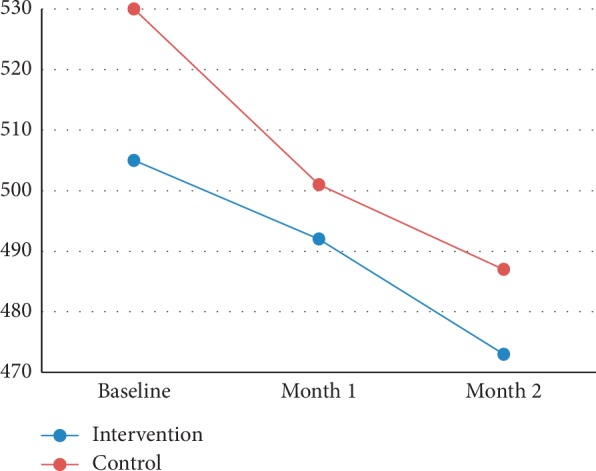
Trend of CMT change in both groups during the study.

Quantitative assessment of CMV and CMT by OCT showed that the decline in CMV and CMT between the groups was insignificant during the study (Tables 2 and 3). Figures 2 and 3 show the trend of CMV and CMT change in both groups during the study (Figures 2 and 3).

### 3.3. Adverse Effects

Mean IOP changes were insignificant in both groups. It changed from 13.47 ± 2.6 at baseline to 14.1 ± 3.1 (*P*=0.53) in the combination group and from 14.8 ± 2.6 to 14.2 ± 3.7 (*P*=0.64) in the IVB alone group, at 3 months, respectively.

In this study, no major ocular complications or systemic side effects were noted regarding IVB and dorzolamide. The severe and intolerable adverse effects of the study did not force any patients to withdraw from the study.

## 4. Discussions

This trial showed that adding topical dorzolamide to IVB did not affect the treatment of DME in the short term. This adjuvant protocol was not successful in the management of our cases in terms of both functional (BCVA) and anatomical (CMV and CMT) outcomes.

Previous studies have described the positive effect of topical dorzolamide eye drops on Cystoid Macular Edema (CME) in patients with RP [11, 12], Usher's Syndrome [12], choroideremia [13], hydroxychloroquine retinopathy [14], macular edema in the early phase after vitrectomy and epiretinal membrane removal [15], retinal vein occlusions [16], and treatment of neovascularization in Age-Related Macular Degeneration (ARMD) [17].

On the one hand, it was clear that the outflow through the anterior chamber may have a role in anti-VEGF clearance [19, 20]. On the other hand, dorzolamide had aqueous suppressant activity [21]. Thus, one possible mechanism for the efficacy of dorzolamide combined with IVB is due to the fact that aqueous suppressant activity of dorzolamide can decrease the wash-out time of anti-VEGF agents [21, 22]. Also, dorzolamide may reduce edema by Muller cell activity and retinal pigment epithelial pump function modulation leading to the fluid regress from the retina to the choroid [23, 24].

The obtained results are not coupled with previous results in the use of topical dorzolamide combined with IVB [16, 17]. The result of the study of Sridhar et al. suggests that the use of topical dorzolamide-timolol with intravitreous anti-VEGF may reduce the central subfield thickness and subretinal fluid in eyes with persistent exudation [17]. The main differences between the current study and the study of Sridhar et al. were the previous use of intravitreous anti-VEGF in which all eyes had been receiving long-term anti-VEGF therapy before study enrollment for a mean of 21.9 injections [17].

In another study, Obeid et al. suggest a potentially beneficial effect of dorzolamide-timolol in eyes with macular edema secondary to retinal vein occlusion resistant to anti-VEGF therapy [16]. Unlike our study, some researchers use a combination of both two aqueous suppressants including dorzolamide and timolol; hence, it is likely that the improvement noted in their study may have been related to the concomitant use of timolol as another aqueous suppressant agent or initiation of aqueous suppressant before the anti-VEGF therapy.

In our study, we exclude patients with the previous history of cataract surgery. So, it should be considered that the intraocular transferability of topical dorzolamide in these eyes is low.

There were some limitations to this study including the small sample size and short duration of dorzolamide treatment. The importance of this pilot study lies in observing the functional and anatomical effect of dorzolamide treatment combined with IVB in DME, which was not been studied before as was conventionally used to treat other causes of ME.

## 5. Conclusion

The result of our study demonstrates that the treatment of ME in diabetic patients with a combination of topical dorzolamide 2% and IVB had no beneficial effects in the treatment of DME at least in a short time. Since there are inconsistent data to use topical dorzolamide as an adjunct to anti-VEGF, larger prospective trials and a combination of timolol-dorzolamide are necessary to evaluate the real effect of topical aqueous suppressants as an adjunct to anti-VEGF therapy in the treatment of DME.

## Figures and Tables

**Table 1:** BCVA logMAR after intervention for both groups.

Time	Control		Intervention		*P*_1_
	Mean	SD	Mean	SD	
Baseline	0.181	0.0911	0.213	0.0806	0.270
Month 1	0.206	0.1063	0.225	0.0931	0.539
Month 2	0.213	0.0957	0.231	0.0946	0.642
*P*_2_	0.115		0.247		

SD: standard deviation, *P*_1_: between-group *P*value (Mann–Whitney test), *P*_2_: within-group *P*value (Friedman test).

**Table 2:** CMV after intervention for both groups.

Time	Control		Intervention		*P*_1_
	Mean	SD	Mean	SD	
Baseline	11.22	1.27	11	1.02	0.780
Month 1	11.03	1.23	10.83	1.07	0.590
Month 2	10.95	1.24	10.71	0.99	0.616
*P*_2_	0.039		0.002		

SD: standard deviation, *P*_1_: between-group *P*value (Mann–Whitney test), *P*_2_: within-group *P*value (Friedman test).

**Table 3:** CMT after intervention for both groups.

Time	Control		Intervention		*P*_1_
	Mean	SD	Mean	SD	
Baseline	530	80	505	87	0.341
Month 1	501	91	492	85	0.696
Month 2	487	108	473	86	0.491
*P*_2_	0.003		0.013		

SD: standard deviation, *P*_1_: between-group *P*value (Mann–Whitney test), *P*_2_: within-group *P*value (Friedman test).

## Data Availability

The data sets used and/or analyzed during the present study are available from the corresponding author on reasonable request.
